# Possible Involvement of Intracellular Calcium-Independent Phospholipase A_2_ in the Release of Secretory Phospholipases from Mast Cells—Increased Expression in Ileal Mast Cells of Crohn’s Disease

**DOI:** 10.3390/cells8070672

**Published:** 2019-07-03

**Authors:** Ulrika Christerson, Åsa V. Keita, Martin E. Winberg, Johan D. Söderholm, Christina Gustafson-Svärd

**Affiliations:** 1 Department of Chemistry and Biomedical Sciences, Faculty of Health and Life Sciences, Linnaeus University, 391 82 Kalmar, Sweden; 2 Department of Clinical and Experimental Medicine, Division of Surgery, Orthopedics & Oncology, Linköping University, 581 85 Linköping, Sweden; 3 Department of Surgery, County Council of Östergötland, 581 85 Linköping, Sweden

**Keywords:** phospholipases A_2_, mast cells, Crohn’s disease, inflammation

## Abstract

Increased activity of secretory phospholipases A_2_ (sPLA_2_) type-II was previously observed in ileum of Crohn’s disease (CD). Our aims were to explore the involvement of calcium-independent (i)PLA_2_β in the release of sPLA_2_s from the human mast cell (MC) line (HMC-1) and investigate expressions of cytosolic (c)PLA_2_α, iPLA_2_β, sPLA_2_-IIA and sPLA_2_-V in MCs of CD ileum. The release of sPLA_2_ was investigated in HMC-1 by immunocytochemistry and ELISA. The expression intensities of PLA_2_s in mucosal MCs, and the proportion of PLA_2_-positive MCs, were investigated in normal ileum and in ileum from patients with CD by immunohistochemistry. The calcium ionophore-stimulated release of sPLA_2_-IIA and sPLA_2_-V from HMC-1 was reduced by the iPLA_2_-inhibitor bromoenol lactone. All four PLA_2_s were detectable in mucosal MCs, both in normal ileum and in CD, but the proportion of iPLA_2_β-containing mucosal MCs and the expression intensity of sPLA_2_-IIA was increased in CD. Results indicate that iPLA_2_β is involved in the secretion of sPLA_2_s from HMC-1, and suggest that iPLA_2_β-mediated release of sPLA_2_ from intestinal MCs may contribute to CD pathophysiology. Ex vivo studies on isolated mucosal mast cells are however needed to clarify the precise role of MC PLA_2_s in the inflammatory processes of CD.

## 1. Introduction

Mediators released from activated intestinal mast cells (MCs) have shown to be of pathophysiological significance in Crohn’s disease (CD) [1,2], for instance, by promoting intestinal fibrosis or by decreasing the mucosal barrier against immune-activating antigens [3,4,5]. However, MC mediators do not necessarily have only detrimental effects in CD, since intestinal MCs also are thought to have a role in host defense against bacterial, viral and parasitic agents [5]. MC mediators of potential relevance for inflammatory conditions include, for instance, eicosanoids [6] and other lipid mediators (i.e., platelet-activating factor and lysophospholipids) generated upon activation of one or several isoforms of the phospholipase A_2_ superfamily (PLA_2_) [7]. The expression of different PLA_2_ isoenzymes in MCs of the human intestinal mucosa is still unknown, both in the normal intestine and in CD. 

The PLA_2_s constitute a superfamily of intracellular and secretory isoenzymes that catalyzes hydrolysis of the *sn*-2 ester of glycerophospholipids, thereby producing free fatty acids and lysophospholipids [7]. The high molecular weight intracellular PLA_2_s, cytosolic PLA_2_ (cPLA_2_; also named group IV PLA_2_) and calcium-independent PLA_2_ (iPLA_2_; also named group VI PLA_2_), are ubiquitously expressed in most tissues and cell types [7]. Among the six different cPLA_2_s known, cPLA_2_α (also named group IVA cPLA_2_) is by far the most studied and evaluated [8]. cPLA_2_α shows marked preference for arachidonic acid (AA) over other fatty acids [8] and is activated by an increase in cytosolic free calcium and phosphorylation [8]. Since cPLA_2_α is AA-specific, it is generally assumed to be the major contributor to the production of inflammatory eicosanoids [8]. In contrast to cPLA_2_, iPLA_2_ shows no strict AA specificity [9], and does not require calcium for its enzymatic activity. iPLA_2_ has been suggested to have diverse biological functions [9], including release of AA for eicosanoid production [9,10] and participation in various neurodegenerative disorders and inflammatory responses [9]. Until today seven iPLA_2_s have been identified, iPLA_2_β (also named group VI-1 and 2 iPLA_2_) being the most widely evaluated [7,9]. Most interesting, a recent study on mice [11] showed that iPLA_2_β deficiency increased colitis severity and ileal damage in DSS-induced colitis, suggesting a protective role for iPLA_2_β in the intestinal mucosa. Indeed, this study [11] points to the importance of further investigations concerning the specific roles of individual PLA_2_ isotypes in inflammatory bowel disease (IBD) [12]. To date, no studies on iPLA_2_ expression or activity in human MCs have been reported. 

The mammalian secretory PLA_2_s, (sPLA_2_s) constitute a group of at least eleven different low molecular weight isoforms [13]. They are all Ca^2+^-dependent and show no apparent fatty acid selectivity [7,13,14]. Individual sPLA_2_s exhibit unique tissue and cellular localizations and their expression varies among species [14]. sPLA_2_s have been investigated in several studies on rodent MCs [15,16,17], whereas only a few studies on sPLA_2_s in human MCs have been reported so far [18,19,20]. sPLA_2_s released to the environment are thought to act in both an autocrine and a paracrine manner [14], and the resulting cellular activities have frequently been associated with various inflammatory conditions [14]. However, the sPLA_2_s have several diverse functions and in addition to their proposed inflammatory actions they seem to have protective and anti-inflammatory functions as well [14,21,22]. Although sPLA_2_s release fatty acids from glycerophospholipids, generating lysophospholipids and AA for eicosanoid synthesis [14,23], they may also act by receptor-mediated, non-catalytic, mechanisms [14]. 

Rodent MCs have shown to express several different sPLA_2_s, including the two closely related isotypes sPLA_2_-IIA and sPLA_2_-V [24], but it is still not known which particular sPLA_2_s are expressed by human intestinal mucosal MCs. If released from mucosal MCs, however, it seems reasonable to believe that sPLA_2_s may, in one way or another, participate in modulating the inflammatory process of the intestinal CD mucosa. It is important, therefore, to investigate which particular sPLA_2_s are present in MCs of the human intestinal mucosa and how the release of these sPLA_2_s is regulated. Since iPLA_2_ has shown to participate in processes related to exocytosis and release of enzymes [10,25,26,27,28] it is relevant to investigate if this PLA_2_ is implicated also in the release of sPLA_2_s from MCs.

The aims were to explore the possible involvement of iPLA_2_β in the release of sPLA_2_s from human MCs using a human MC line (HMC-1) [29] and to investigate the expressions of cPLA_2_α, iPLA_2_β, sPLA_2_-IIA and sPLA_2_-V in mucosal MCs from normal and CD ileum. 

## 2. Materials and Methods

### 2.1. Cell Culture

The human leukemia MC line-1, HMC-1 [29], was a kind gift from Dr. J.H Butterfield, Mayo Clinic, MN. Cells were cultured in Iscove’s Modified Dulbecco’s Medium (IMDM) (Gibco BRL, Gaithersburg, MD, USA) supplemented with 100 µg/mL streptomycin (Gibco), 100 U/mL penicillin (Gibco), 10% fetal bovine serum (Gibco), and 1.2 mM α-thioglycerol (Sigma-Aldrich, St. Louis, MO, USA) and kept in a humidified atmosphere with 5% CO_2_ at 37 °C. Cell viability was routinely evaluated by the trypan blue exclusion assay or by a MTT toxicology assay and was not affected during the experimental conditions used in this study.

To investigate if the expressions of iPLA_2_β and cPLA_2_α could be further increased upon activation of the MCs, 5 × 10^5^ HMC-1 were incubated for 48h in 1 mL culture medium with or without (controls) 25 ng/mL of TNFα. TNFα is of fundamental importance in inflammatory conditions such as CD [30], and may influence PLA_2_ expression and activity [31,32]. The expressions of cPLA_2_α, iPLA_2_β, sPLA_2_-IIA and sPLA_2_-V were after incubation analyzed by Reverse Transcriptase-PCR and immunocytochemical staining.

### 2.2. Reverse Transcriptase-PCR of PLA_2_s

Total RNA was extracted from HMC-1 using Ultraspec™-II RNA Isolation System (Nordic Biosite, Täby, Sweden). One µg of total RNA was converted into cDNA using Omniscript^®^ Reverse Transcription RT Kit (Qiagen, Solna, Sweden) according to the manufacturer’s instructions, and amplified using PuRe Taq RTG PCR beads (GE Healthcare, Buckinghamshire, UK) and primers (Life Technology Ltd., Paisley, UK). Due to a high expression, the cDNA for sPLA_2_-IIA had to be diluted 10× before subjected to conventional Reverse Transcriptase-PCR. Primers and running schedules used in PCR are summarized in Table 1. The final PCR products were loaded on 1.5% agarose gels, and identified as previously described [33]. 

### 2.3. Immunocytochemical Staining of PLA_2_s

HMC-1 were smeared on poly-*L*-lysine coated glass (Sigma) as previously described [33]. The samples were fixed in ice-cold acetone for 5 min at –20 °C and then blocked with 50% of serum in PBS for 1h at room temperature (RT). The samples were incubated with either 1:50 mouse monoclonal FITC-conjugated anti-human sPLA_2_-V antibody (Santa Cruz, Dallas, Texas, USA) or 1:200 mouse monoclonal anti-human sPLA_2_-IIA (Cayman Chemical Co, Ann Arbor, MI, USA) for 16h at 4 °C. Biotin-conjugated 1:250 secondary rabbit anti-mouse (DakoCytomation, Glostrup, Denmark) was applied to samples with sPLA_2_-IIA antibody for 1h at RT and then 1:100 FITC-conjugated streptavidin (DakoCytomation) for 30 min at RT. In addition, samples were incubated with either 1:100 Alexa-488 conjugated mouse monoclonal anti-human cPLA_2_α (Santa Cruz) or 1:250 rabbit polyclonal anti-human iPLA_2_β (Cayman) for 16h at 4 °C. FITC-conjugated secondary antibody goat anti-rabbit (Jackson ImmunoResearch Laboratories Inc, West Grove, PA, USA) was applicated at a dilution of 1:400. The slides were mounted with Vectashield^®^ mounting medium with propidium iodide (Vector Laboratories Inc, Burlingame, CA, USA). Negative controls without primary antibodies or with a FITC-conjugated isotype matched irrelevant antibody (Santa Cruz) were included in all experiments.

### 2.4. Release of Fatty Acids

To further explore the involvement of cPLA_2_ in AA-mobilization in activated HMC-1, ^14^C-AA labelled cells were stimulated with the frequently used MC activator calcium ionophore A23187 [10,34,35,36,37,38], in the presence and absence of known enzyme inhibitors. Cells were suspended in 25 mL supplemented medium with 0.1% fatty-acid free bovine serum albumin (Sigma) and labelled for 16 h with 0.1 µCi [1-^14^C]AA (New England Nuclear, Perkin Elmer, Wellesley, MA, USA) per 5 × 10^5^ cells, before washed two times with PBS supplemented with 0.1% fatty-acid free bovine serum albumin [37]. Labelled cells (5 × 10^5^ cells in a final volume of 2.7 mL) were then treated for 4h with 2 μM of the calcium ionophore A23187 (Sigma) only, or in combination with 200 nM of the protein kinase C activator phorbol myristate acetate (PMA) (Sigma). The combination of A23187 and PMA has previously shown to induce a synergistic release of AA in other cell systems, an effect attributed to an increased activation of cPLA_2_ [37,39,40]. 

As an attempt to investigate the relative contribution of cPLA_2_ and iPLA_2_ in the A23187-stimulated AA release, cells were pre-incubated with the combined cPLA_2_ and iPLA_2_ inhibitor methyl arachidonyl fluoro-phosphonate (MAFP) (Sigma) [41], or the specific iPLA_2_ inhibitor bromoenol lactone (BEL) (Sigma) [41]. Cells were pre-treated for 30 min with MAFP (0 μM, 10 μM or 25 μM) or BEL (0 μM, 10 μM or 25 μM) prior to incubation with A23187 (2 µM) for an additional 4 h. All treatments with stimulators and inhibitors were performed in the absence of serum but in the presence of 0.1% fatty acid-free bovine serum albumin. The amount of ^14^C-AA released into the culture medium was analyzed by liquid scintillation counting. The inhibitors were added 30 min prior to adding the stimulators. To evaluate the AA specificity of the involved PLA_2_, a comparable stimulation of ^14^C-oleic acid (OA) (Perkin Elmer) labelled cells was performed. 

### 2.5. Degranulation and Release of sPLA_2_

Cellular events leading to an increased cytosolic Ca^2+^ concentration may stimulate degranulation of MCs [42]. Therefore, we next investigated if sPLA_2_-IIA and V were released from A23187-stimulated HMC-1. HMC-1 (5 × 10^5^ cells in a final volume of 150 µL) were treated with A23187 (0 μM, 1 μM, 2 μM, 4 μM) for 4 h. 

To investigate if iPLA_2_ is involved in the ionophore-stimulated sPLA_2_ secretion in HMC-1, 25 µM of the inhibitor BEL was added 30 min before A23187, when appropriate. All treatments with stimulators and inhibitors were performed in the absence of serum. Cells were centrifuged and the medium was collected. The β-hexosaminidase activity was determined as previously described [43], and the amount of sPLA_2_-IIA was determined by sandwich-ELISA according to the manufacturer´s instructions (Cayman). 

The amounts of remaining sPLA_2_-IIA and sPLA_2_-V in stimulated cells were investigated by immunocytochemical staining as described above. Due to its low basal expression, sPLA_2_-V had to be upregulated by 25 ng/mL TNFα (Sigma) for 48h prior to stimulation with A23187 in this set of experiments.

### 2.6. Patients 

Specimens from ileum were achieved during surgery at Linköping University Hospital from 5 patients with ileal CD and 5 patients with colonic cancer, as non-IBD controls. The CD patients constituted of 3 men and 2 women with a median age of 53 years (range 43–65) and disease duration of 15 years (range 9–28). According to the Montreal classification, all patients had an active disease, however, tissue obtained for analyzes were dissected from mild-inflamed ileum. The non-IBD control group constituted of microscopically normal ileal specimens from 3 men and 2 women with a median age of 71 years (range 62–87). None of the patients within the non-IBD control group had received pre-operative chemo- or radiotherapy or had signs of generalized disease. The study was approved by the Committee of Human Ethics, Linköping (ethical number 02-154, 09/04/2002) and all included subjects gave their informed written consent before the study was initiated. 

### 2.7. Preparation of Ileal Tissues

Surgical ileal specimens from patients with CD and non-IBD controls were immediately after division of the ileocolic artery, put in ice-cold oxygenated Krebs buffer and specimens were stripped of external muscle and myenteric plexus, as previously described [44]. Segments of ileal mucosa were fixed in 4% buffered formaldehyde in PBS for 24h in 4 °C, embedded in paraffin and sectioned to a thickness of 5 µm.

### 2.8. Immunohistochemical Staining of PLA_2_s

Slides with sections were hydrated according to standard procedures followed by incubation for 10 min with background sniper (Histolab, Gothenburg, Sweden). After washed in PBS, slides were incubated for 16h at 4 °C with 1:200 mouse monoclonal-anti-human MC tryptase antibody (Santa Cruz) in combination with either 1:50 rabbit polyclonal-anti-human sPLA_2_-IIA (Novus Biologicals, Bio-Techne, Abingdon, UK), 1:50 rabbit polyclonal-anti-human sPLA_2_-V (Bio-Techne), 1:50 goat polyclonal cPLA_2_β antibody (Santa Cruz), or 1:50 rabbit polyclonal-anti-human iPLA_2_β (Santa Cruz). Slides were rinsed and incubated with secondary antibodies (MC: 1:4 ready to use Alexa Fluor 594-conjugated-goat-anti-mouse (Invitrogen, Oregon, USA); cPLA_2_β: 1:200 Alexa Fluor 488-conjugated donkey-anti-goat (Life technologies); iPLA_2_β, sPLA_2_-IIA, sPLA_2_-V: 1:200 Alexa Fluor 488-conjugated donkey-anti-rabbit (Life technologies) for 1h at RT. After repeated rinsing, slides were mounted with Prolong^®^ Gold Antifade with DAPI (Life Technologies) and evaluated in a Nikon E800 fluorescence microscope connected to software NIS elements (Nikon Instruments Inc. Tokyo, Japan) in a blinded fashion by two independent researchers. Three sections per individual were stained for each double-staining, and negative controls with primary antibodies excluded were included in all experiments. The total number of MCs co-localizing with the different PLA_2_s were manually quantified at 600× magnification. The intensities of the different PLA_2_-stainings were measured by Image J Fiji software (National Institutes of Health, Bethesda, MD, USA). Approximately 6–8 area-units per section were counted. All area-units were of the same size and only area-units that were fully covered by tissue were used. 

### 2.9. Statistical Analysis

Data were analyzed using the GraphPad Prism Software (GraphPad Software Inc., CA, USA). Parametric data are expressed as mean ± SEM and depending on the experimental layout, statistical analyses were undertaken with one-way ANOVA, repeated measures ANOVA, and Bonferroni post-test. Non-parametric data are given as median (25th–75th interquartile range) and comparisons between groups were done with Kruskal-Wallis and Mann-Whitney U tests.

## 3. Results

### 3.1. iPLA_2_ is the Predominating High-Molecular-Weight PLA_2_ Expressed by HMC-1

**Figure 1:**
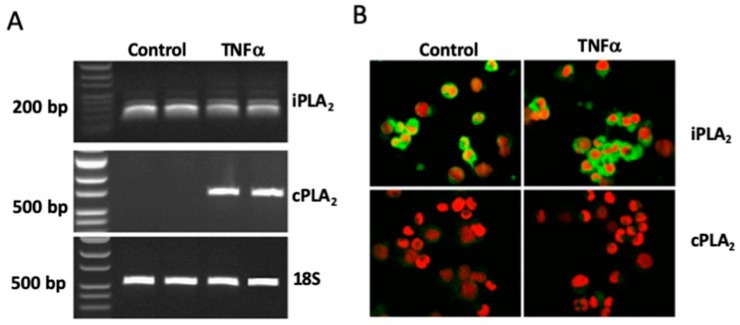
Expression of iPLA_2_β and cPLA_2_α in HMC-1. Cells were stimulated with TNFα (25 ng/mL) or culture medium (control) for 48 h. (**A**) Reverse Transcriptase-PCR analysis; PCR products were identified as iPLA_2_α (184 bp), cPLA_2_β (737 bp) or 18S rRNA (531 bp). Results are presented as duplicate samples representative of three independent experiments. (**B**) Immunocytochemical analysis; green staining is for either iPLA_2_β or cPLA_2_α. Cell nuclei were visualized with propidium iodide staining (red) (magnification × 600). Results are representative for three independent experiments.

HMC-1 was found to have a basal expression of both iPLA_2_β mRNA (Figure 1A) and iPLA_2_β protein (Figure 1B). In contrast, cPLA_2_α revealed no basal mRNA expression (Figure 1A), and the protein expression was very low (Figure 1B). Treatment with 25 ng/mL TNFα for 48 h did neither affect the iPLA_2_β mRNA expression (Figure 1A) nor the iPLA_2_β protein expression (Figure 1B). On the contrary, TNFα stimulation had an inconsistent effect on the cPLA_2_α expression, increasing the mRNA stimulation had an inconsistent effect on the cPLA_2_α expression, increasing the mRNA expression (Figure 1A) without affecting the protein expression (Figure 1B).

### 3.2. Secretory PLA_2_-IIA and V are Expressed by HMC-1

**Figure 2:**
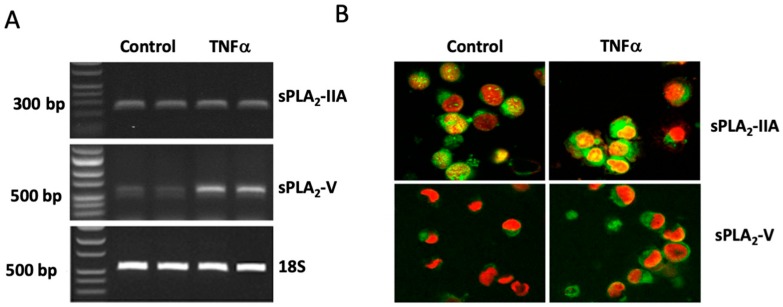
Expression of sPLA_2_-IIA and sPLA_2_-V in HMC-1. Cells were either stimulated with TNFα (25 ng/mL) or culture medium (control) for 48 h. (**A**) Reverse Transcriptase-PCR analysis; the PCR products were identified as sPLA_2_-IIA (238 bp), sPLA_2_-V (559 bp) or 18S rRNA (531 bp). Note that the cDNA for sPLA_2_-IIA was diluted ten times compared to the cDNA for sPLA_2_-V. Samples are two representatives out of seven independent runs. (**B**) Immunocytochemical analysis. Green staining is for sPLA_2_-IIA or sPLA_2_-V and red staining is for visualization of cell nuclei (magnification × 600). Results are representative for three independent experiments.

Immunostaining revealed a basal expression of sPLA_2_-IIA mRNA (Figure 2A) and sPLA_2_-IIA protein (Figure 2B) in HMC-1. Neither the mRNA nor the protein expression was affected in cells stimulated with 25 ng/mL TNFα for 48h (Figure 2A,B). HMC-1 were also found to have a basal expression of sPLA_2_-V mRNA (Figure 2A) and sPLA_2_-V protein (Figure 2B), although, less pronounced as compared to corresponding expressions of sPLA_2-_IIA (Figure 2A,B). However, in contrast to sPLA_2_-IIA, the expressions of sPLA_2_-V mRNA and proteins were increased in TNFα-stimulated cells (Figure 2A,B).

### 3.3. cPLA_2_α is not Involved in Calcium Ionophore-Stimulated AA Mobilization in HMC-1

**Figure 3:**
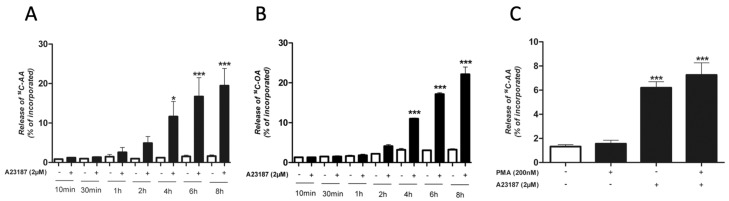
Release of radiolabeled fatty acids from A23187-stimulated HMC-1 cells. Control cells were incubated with culture medium only. (**A**) Time-dependent release of arachidonic acid (AA). (**B**) Time-dependent release of oleic acid (OA). (**C**) Effect of combined stimulation with calcium ionophore A23187 and phorbol myristate acetate (PMA) on the release of AA. PMA and/or A23187 were added for 4 h. * *p* < 0.05, ** *p* < 0.01, *** *p* < 0.001 vs. control cells. Data from three independent experiments.

Stimulation with calcium ionophore A23187 caused an obvious time-dependent increase in the release of radioactivity from ^14^C-AA-labelled cells (Figure 3A). The increase was discernible after 1 h but not significant until 4 h of treatment compared to controls at each time point (Figure 3A). cPLA_2_α is generally regarded as the main regulator of cellular AA mobilization [8], however, a comparable release of radioactivity also from A23187-stimulated ^14^C-OA-labeled HMC-1 clearly demonstrated that the ionophore-stimulated PLA_2_ activity was not AA-specific (Figure 3B). Stimulation with the combination of A23187 and the protein kinase C activator PMA showed that PMA had no further impact on the A23187-stimulated AA release, neither at 30 min (data not shown) nor at 4 h (Figure 3C).

**Figure 4:**
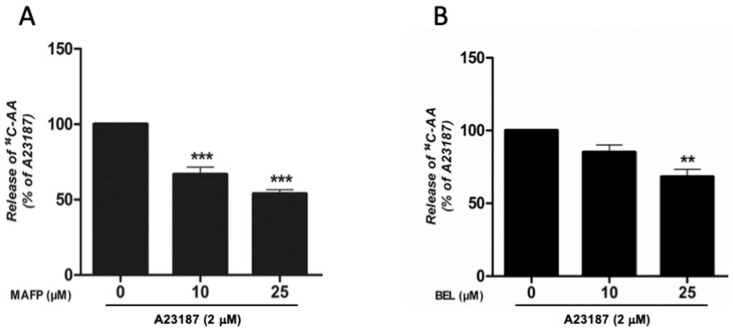
Effect of PLA_2_ inhibitors on the calcium ionophore A23187-stimulated release of radiolabeled arachidonic acid (AA) from HMC-1. Cells were pre-treated for 30 min with various concentrations of PLA_2_ inhibitors, prior to incubation with A23187 (2 µM) for an additional 4 h. (**A**) Effect of the combined cPLA_2_ and iPLA_2_ inhibitor methyl arachidonyl fluoro-phosphonate (MAFP). (**B**) Effect of the specific iPLA_2_ inhibitor bromoenol lactone (BEL). *p < 0.05, ** *p* < 0.01, *** *p* < 0.001 vs. A23187-stimulated cells. Data from three independent experiments.

The PLA_2_-inhibitors MAFP (general) and BEL (iPLA_2_-specific) were found to reduce the A23187-stimulated AA release in a dose-dependent manner and at a comparable extent (Figure 4A,B).

### 3.4. iPLA_2_ is Involved in the A23187-Stimulated Release of sPLA_2_-IIA and sPLA_2_-V from HMC-1

**Figure 5:**
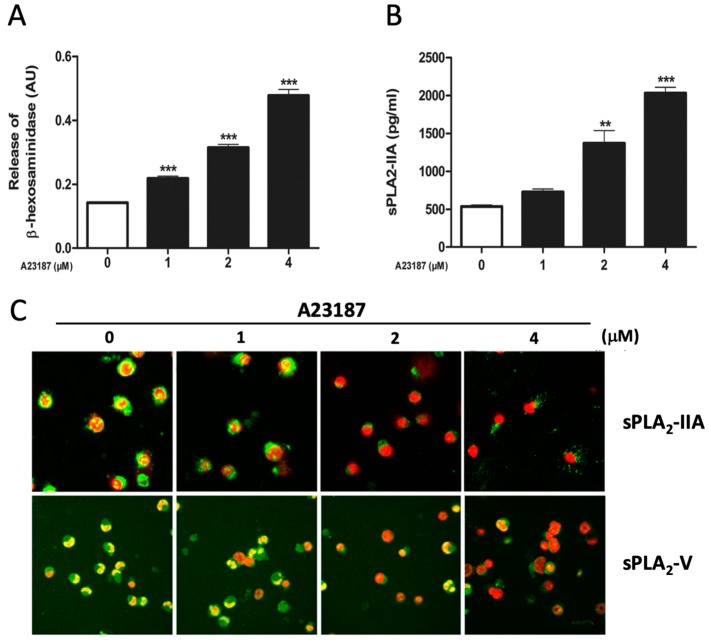
Degranulation and release of sPLA_2_-IIA and sPLA_2_-V in A23187-stimulated HMC-1. Cells were stimulated for 4 h with various concentrations of calcium ionophore A23187. Control cells were incubated with culture medium only. (**A**) Release of β-hexosaminidase. (**B**) ELISA analysis. Release of sPLA_2_-IIA. (**C**) Immunocytochemical analysis, visualizing the effect of A23187 on the release of sPLA_2_-IIA and sPLA_2_-V. Green staining is for sPLA_2_-IIA or sPLA_2_-V and red staining is for visualization of cell nuclei (magnification × 600). Note that the expression of sPLA_2_-V had to be upregulated by TNFα, as described in Figure 2A and B. **p < 0.01, ***p < 0.001 vs. controls. Data from three independent experiments.

Stimulation with A23187 induced degranulation of the HMC-1 cells in a dose-dependent manner, demonstrated as an increased β-hexosaminidase release (Figure 5A). Simultaneously, A23187 caused a dose-dependent release of sPLA_2_-IIA, as detected by ELISA (Figure 5B) and further confirmed by immunocytochemical visualization (Figure 5C). In addition, A23187 caused a dose-dependent release of sPLA_2_-V, as visualized by immunocytochemistry (Figure 5C). Due to the low basal expression of sPLA_2_-V, the immunocytochemistry was performed after up-regulation of sPLA_2_-V with TNFα, as illustrated in Figure 2B. 

**Figure 6:**
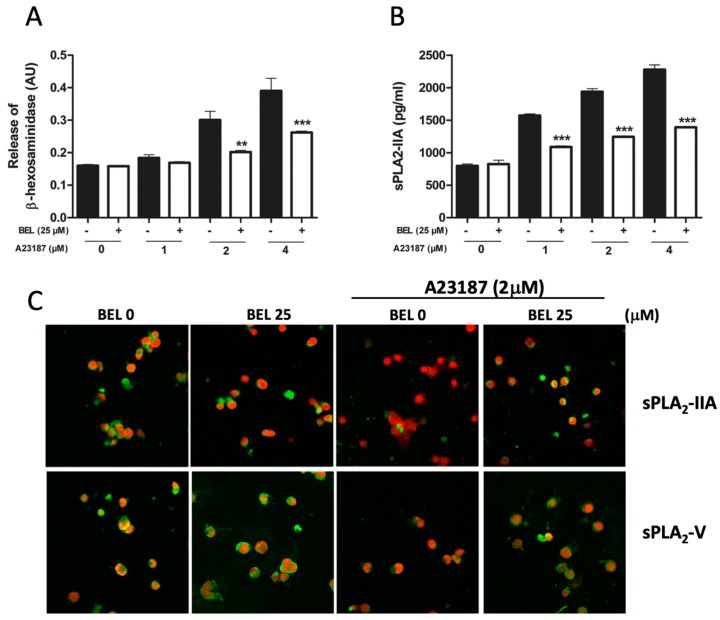
Effect of iPLA_2_ inhibition on A23187-induced degranulation and release of sPLA_2_-IIA and sPLA_2_-V in HMC-1. (**A**) Effect of the specific iPLA_2_ inhibitor bromoenol lactone (BEL) on the release of β-hexosaminidase. (**B**) Effect of the specific iPLA_2_ inhibitor BEL on the release of sPLA_2_-IIA measured by ELISA. (**C**) Immunocytochemical analysis, visualizing the effect on the release of sPLA_2_-IIA and sPLA_2_-V. Green staining is for sPLA_2_-IIA or sPLA_2_-V and red staining is for visualization of cell nuclei (magnification × 600). Note that the expression of sPLA_2_-V had to be upregulated by TNFα, as described in Figure 2A and B, to be illustrated. ** *p* < 0.01, *** *p* < 0.001 vs. controls. Data from three independent experiments.

Pre-incubation with the iPLA_2_-specific inhibitor BEL prior to A23187 stimulation, diminished both the degranulation of HMC-1 (Figure 6A) and the release of sPLA_2_-IIA and sPLA_2_-V (Figure 6B,C).

### 3.5. Mucosal MCs Express all four PLA_2_ Isoforms Investigated

**Figure 7:**
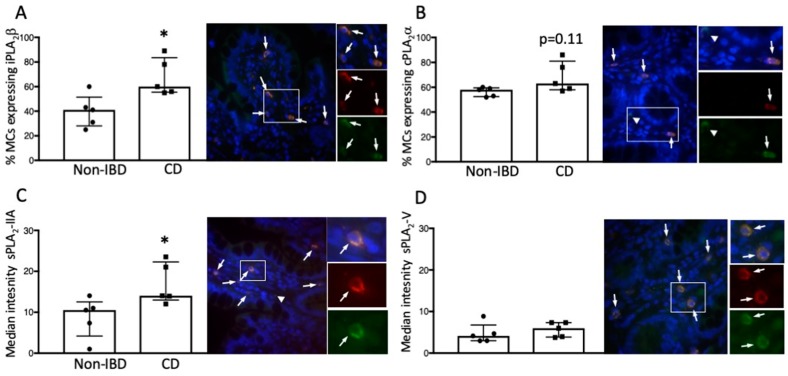
Expression of iPLA_2_β, cPLA_2_α, sPLA_2_-IIA and sPLA_2_-V on mast cells (MCs) in the intestinal mucosa of 5 patients with Crohn’s disease (CD) and 5 controls. (**A**) Percentage of MCs expressing iPLA_2_β. Arrows indicate MCs co-localizing with iPLA_2_β in a patient with CD. (**B**) Percentage of MCs expressing cPLA_2_α. Arrows indicate MCs co-localizing with cPLA_2_α in a control patient. Arrow-head indicates cPLA_2_α expression in a cell not positive for MC tryptase. (**C**) Expression intensity of sPLA_2_-IIA on MCs. Arrows indicate MCs co-localizing with sPLA_2_-IIA in a patient with CD. Arrow-head indicates sPLA_2_-IIA expression in a cell not positive for MC tryptase. (**D**) Expression intensity of sPLA_2_-V on MCs. Arrows indicate MCs co-localizing with sPLA_2_-V in a control patient.

Cells positively stained with the MC tryptase antibody were found in both control and CD ileal mucosa. MCs from controls and CD patients were found to express all four PLA_2_ isoforms investigated, i.e., the two intracellular high molecular isoforms, cPLAα and iPLA_2_β, and the two secretory isoforms, sPLA_2_-IIA and sPLA_2_-V (Figure 7A–D). Both intracellular and secretory PLA_2_s were also found on cells not positive for MC tryptase, and in addition, there were MCs present not expressing any PLA_2_.

MC and PLA_2_ expressions were quantified manually at 600× magnification and results are given as median (25th–75th percentile). Red = MCs, Green = PLA_2_, Blue = DAPI, nuclei staining. *p < 0.05 vs. controls.

### 3.6. Increased Proportion of iPLA_2_β-Containing Mucosal MCs of CD Ileum

For the intracellular forms there was a higher percentage of MCs expressing iPLA_2_β in CD compared to controls, *p* < 0.05 (Figure 7A), but no significant difference in expressions of cPLA_2_α, *p* = 0.11 (Figure 7B). Measurements of intensity (Median (25th–75th percentile)) showed no difference between the groups (iPLA_2_β: CD 13.1 (12.1–16.3); non-IBD 12.9 (11.2–14.5), *p* = 0.69, and cPLAα; CD 21.8 (18.1–32.3); non-IBD 17.4 (14.2–20.9), *p* = 0.22). 

### 3.7. Increased Expression Intensity of sPLA_2_-IIA in Mucosal MCs of CD Ileum

For the secretory PLA_2_s, there was no difference in the percentage of MCs expressing either sPLA_2_-IIA (Non-IBD 71.0% (52.3–74.1); CD 69.0 (48.5–73.5)) or sPLA_2_-V (Non-IBD 37.0 (25.5–57.5); CD 50.0 (30.0–50.1)). In contrast, intensity measurements showed a significantly higher expression intensity of sPLA_2_-IIA in MCs of CD patients compared to controls, *p* < 0.05 (Figure 7C), but no difference between groups in the expression of sPLA_2_-V (Figure 7D). 

## 4. Discussion

The present study demonstrates, for the first time, that human ileal MCs of normal and CD mucosa contain the sPLA_2_ isoforms sPLA_2_-IIA and sPLA_2_-V, as well as the intracellular high molecular isoforms cPLA_2_α and iPLA_2_β. In addition, studies on the human MC cell line HMC-1 demonstrated that iPLA_2_β might have a role in the release of sPLA_2_-IIA and sPLA_2_-V. Thus, our results point to a possible role of iPLA_2_β in the release of sPLA_2_s from MCs of the human ileal mucosa. 

sPLA_2_-IIA and V are frequently associated with inflammatory conditions [14,23]. Even though sPLA_2_-II is known to be present in the CD intestine [45,46], including submucosal MCs [18], no studies on sPLA_2_-V expressions in CD intestine, or sPLA_2_-II expressions in intestinal mucosal MCs, have been reported. We previously demonstrated [46] that the distal ileal mucosa is rich in PLA_2_-II mRNA and that the expression of this mRNA and the corresponding enzyme activity accompanies recurrent new ileal inflammation after ileocolonic resection for CD. However, the cells responsible for this increased expression and activity have previously not been identified. In the present study we demonstrated that the expression of sPLA_2_-IIA was higher in MCs from ileal CD mucosa compared to MCs from control patients. Further, we found that the proportion of iPLA_2_β-expressing mucosal MCs was increased in CD ileum compared with controls; i.e., among all MCs present, more MCs expressed iPLA_2_β in ileum from CD patients. These findings suggest that MCs may contribute to the increased sPLA_2_-II expression and activity in CD ileum [46].

Although iPLA_2_β is generally thought to be involved in various cellular and pathological conditions [9], its expression and role in the human intestine has never been investigated. However, our results on HMC-1 support previous findings demonstrating a possible role for iPLA_2_β in MC exocytosis [10], and one might speculate that the increased proportion of iPLA_2_β-expressing MCs found in CD may reflect a greater release of various MC mediators in the CD intestine. Intestinal barrier dysfunction, leading to increased transfer of luminal bacteria to the lamina propria is thought to be a factor of importance in the pathogenesis of CD [47]. Considering the proposed protective role of iPLA_2_β in the intestine [11,12], it is tempting to speculate that iPLA_2_β might have a role in releasing bactericidal sPLA_2_s from MCs in the intestinal mucosa. Indeed, several sPLA_2_s, in particular sPLA_2_IIA, are known to have antibacterial activities [7,13,21]. 

Considering the proposed species differences with regard to both MC characteristics [48] and PLA_2_ expression [14] a human experimental MC cell model was used for the studies on sPLA_2_ release. Although various aspects of PLA_2_s have been extensively studied in rodent MCs [10,15,16,17,38,49,50,51], not much is known about the expression and regulation of these enzymes in MCs of human origin. We chose to work with the human MC cell line HMC-1 [29] because it has been frequently used for studies on various aspects of MC biology, and this cell line has been reported to produce several different eicosanoids upon stimulation with calcium ionophore [35,36]. However, the PLA_2_s responsible for generating the required free AA is not known, and studies concerning the expression and activity of PLA_2_-enzymes of HMC-1 are still lacking. It was necessary thus to confirm the presence of intracellular and secretory PLA_2_s in this cell line before using it for studies on sPLA_2_ release. Interestingly the HMC-1 was found to have a basal expression of iPLA_2_β protein, whereas the expression of cPLA_2_α was very low. Neither the protein nor the mRNA expression of iPLA_2_β was apparently affected by TNFα. This lack of effect of TNFα suggests that iPLA_2_β is not regulated by inflammatory agents in HMC-1, a finding well in line with the proposed role of iPLA_2_β as a homeostatic enzyme in cellular phospholipid metabolism [9]. In contrast, TNFα increased the mRNA but not the protein expression of cPLA_2_α. Thus, increasing the level of cPLA_2_α mRNA in HMC-1 seems not to per se induce translation into cPLA_2_α protein, but additional stimulators of translation seem to be needed. These findings are in line with a previous study [52], showing that transforming growth factor β-1 stimulates cPLA_2_ gene expression in human intestinal MCs without affecting the level of cPLA_2_ protein. The translation of gene expressions to protein levels is a multistep process and Schwanhausser et al. [53] has concluded that translational rate constants were the dominant factors in controlling protein levels, and that half-life of the proteins are highly involved in the translation as well. In addition to the findings of increased iPLA_2_β, the HMC-1 were found to have a basal expression of both sPLA_2_-V and sPLA_2_-IIA, which is in line with previous reports on rodent MCs [16] and human lung MCs [20]. However, when HMC-1 were stimulated with TNFα, both mRNA and protein expressions of sPLA_2_-V were increased, whereas the mRNA and protein levels of sPLA_2_-IIA was unaltered. Our results on HMC-1 are in line with previous studies showing that despite close similarities between group IIA and V [14], their expression and regulation may differ [54]. 

The expression of cPLA_2_α protein appears to be very low in HMC-1. Therefore, to clarify if cPLA_2_α activity is present in HMC1, the release of AA and OA was compared in A23187-stimulated cells. The calcium ionophore A23187 caused a marked elevation of fatty acid release from the HMC-1. This fatty acid release was not restricted to AA, and about equally reduced by the specific iPLA_2_ inhibitor BEL [41] and the combined iPLA_2_ and cPLA_2_ inhibitor MAFP [41]. Also, the A23187-stimulated AA release was not augmented by the attempt to increase the cPLA_2_α activity by combined stimulation with PMA [37,39,40]. Taken together, these findings strongly suggest that one or several PLA_2_s, different from the AA specific cPLA_2_α, is accountable for the A23187-stimulated AA release in HMC-1. One possible candidate is iPLA_2_β, since the AA release was reduced by BEL and iPLA_2_ is known to release AA in other cell systems [9,10]. However, BEL and MAFP reduced about 50% of the AA release induced by A23187, indicating contribution of one or several MAFP/BEL-insensitive PLA_2_s, for instance sPLA_2_s [14]. It was out of the scope of the present study to investigate in detail which particular PLA_2_s are involved in the AA release from HMC-1. However, our results may suggest a role for iPLA_2_ and clearly indicate that the cPLA_2_α activity of HMC1 is very low and in line with the low cPLA_2_α protein levels found. 

Whereas several studies have implicated a role for cPLA_2_ and sPLA_2_ in the release of AA from rodent MCs [6,15,17,50,51,55], only one study, so far, has reported involvement of iPLA_2_ [10]. Indeed, A23187 was found to release radiolabeled AA from mouse bone marrow-derived MCs (BMMCs) and rat basophilic leukemia MCs (RBL 2H3) by an iPLA_2_-dependent mechanism [10], a finding in line with our results in HMC-1.

The mechanism of MC degranulation involves cellular events leading to an increased cytosolic Ca^2+^- concentration [42]. Evidently, we found that A23187 stimulates degranulation (i.e., stimulated the release of β-hexosaminidase) of HMC-1 and release of sPLA_2_. This is in line with a previous study on ionophore-stimulated BMMCs [34]. The A23187-stimulated release of sPLA_2_-IIA and sPLA_2_-V was reduced by the iPLA_2_ inhibitor BEL, suggesting a role for iPLA_2_ in the A23187-stimulated sPLA_2_ release from HMC-1. Although BEL is known to inhibit degranulation of BMMCs and RBL 2H3 cells [10], and also to inhibit exocytosis in other cell types [25,28], this is, as far as we know, the first study suggesting a role for iPLA_2_ in the regulation of sPLA_2_ release. Indeed, our finding that BEL inhibited not only the A23187-stimulated release of sPLA_2_, but also the release of β-hexosaminidase, may indicate a role of iPLA_2_ in MC degranulation and release of MC mediators in general. 

Although the results of the present study suggest that iPLA_2_β is involved in the release of sPLA_2_s from A23187-stimulated cells, the precise mechanism by which iPLA_2_β is activated by A23187 has to be evaluated. However, one possible mechanism might be that depletion of calcium stores by A23187 results in displacement of inhibitory calmodulin from iPLA_2_ [49].

Both iPLA_2_β [10,25,26,27,28], and cPLA_2_α [8,56,57] have been implicated in vesicle trafficking and exocytosis. However, due to the low (perhaps absent) cPLA_2_α activity of the HMC-1, it is not likely that cPLA_2_α is involved in the release of sPLA_2_s. Our finding that cPLA_2_α is expressed in human intestinal MCs may suggest, however, that also this intracellular PLA_2_ might be involved in MC exocytosis in the human intestine. Clearly, further studies on MCs isolated directly from the human intestine are needed to evaluate the precise roles of iPLA_2_β and cPLA_2_α in the release of sPLA_2_s from MCs in the normal and inflamed human intestine.

Although our results suggest that iPLA_2_β is involved in the degranulation and release of sPLA_2_ in HMC-1, this is not necessarily true for other experimental MC models or during other experimental settings. For example, a study on BMMCs [38] demonstrated, in contrast with a previous report [10], that iPLA_2_β is not involved in the release of β-hexosaminidase from these MCs. It is also worth mentioning that species differences among MCs may influences their behavior [48], and that it is unknown to what extent the role and regulation of a particular PLA_2_ in rodent MCs correspond to its role and regulation in human MCs. 

BEL is a widely used inhibitor of iPLA_2_, with limited effect on cPLA_2_ and sPLA_2_ [9,41]. Indeed, BEL is to date the only irreversible specific inhibitor of iPLA_2_ available, however, BEL may have other unspecific side effects as well, resulting in cytotoxic effects [58]. In the present study, the viability of HMC-1 was routinely evaluated and no detrimental effect of BEL was found. Thus, it seems likely that iPLA_2_ was the target of BEL in HMC-1. However, to verify this, further studies using gene silencing techniques are needed. 

## 5. Conclusions

In conclusion, this study suggests that iPLA_2_β might be involved in the secretion of sPLA_2_s from HMC-1, suggesting that an iPLA_2_β-mediated release of sPLA_2_ from intestinal MCs may contribute to increased sPLA_2_-II activity. Further, cPLA_2_α, iPLA_2_β, sPLA_2_- IIA and sPLA_2_-V are all present in mucosal MCs of both normal ileum and in the mild-inflamed ileum of CD. However, CD ileum possessed an increased proportion of iPLA_2_β-containing MCs. Taken together, results may suggest that iPLA_2_β may have a previously unrecognized role in human MCs, i.e., regulation of sPLA_2_ secretion. However, further ex vivo studies are needed to confirm this and to evaluate the precise role of iPLA_2_β in the release of sPLA_2_s from isolated ileal MCs and its importance in the pathophysiology of CD.

## Figures and Tables

**Table 1:** Primers and running schedules used in Reverse Transcriptase-PCR.

Gene	Primers (5′ > 3′)	Product (bp)	Running Scheme ^a^
iPLA_2_β	F: AAGGCCTCATCATCATCCAG R: CGGAACACCTCATCCTTCAT	184	40 cycles: 94 °C, 30 s; 60 °C, 30 s; 72 °C, 30 s
cPLA_2_α	F: ATGCCCAGACCTACGATTTA R: AGGGGTTTTCTTCATACTTC	737	40 cycles: 94 °C, 30 s; 55 °C, 30 s; 72 °C, 50 s
sPLA_2_-IIA	F: AAGCCGCACTCAGTTATGG R: GCAGCAGCCTTATCACACT	238	25 cycles: 94 °C, 30 s; 55 °C, 30 s; 72 °C, 30 s
sPLA_2_-V	F: GCTTGGTTCCTGGCTTGTAG R: ACTCGCTGGAGGGTACAGTG	559	30 cycles: 94 °C, 30 s; 55 °C, 30 s; 72 °C, 40 s
18S-rRNA	F: ACGRACCAGAGCGAAAGCAT R: GGACATCTAAGGGCATCACAGAC	531	20 cycles: 94 °C, 20 s; 58 °C, 20 s; 72 °C, 45 s

^a^ The first cycle was preceded by an initial denaturation step at 94 °C for 5 min, and the last cycle was followed by an elongation step at 72 °C for 5 or 7 (cPLA_2_) min.

## Abbreviations

A23187, calcium ionophore; AA, arachidonic acid; BEL, bromoenol lactone; CD, Crohn’s disease; cPLA_2_, cytosolic phospholipase A_2_; IMDM, Iscove’s Modified Dulbecco’s Medium; IBD, inflammatory bowel disease; iPLA_2_, calcium-independent phospholipase A_2_; MAFP, methyl arachidonyl fluoro-phosphonate; MC, mast cell; OA, oleic acid; PLA_2_, phospholipase A_2_; PMA, phorbol myristate acetate; RT, room temperature; sPLA_2_, secretory phospholipase A_2_; sPLA_2_-IIA, secretory phospholipase A_2_ group IIA; sPLA_2-_V, secretory phospholipase A_2_ group V.
